# Evaluation of the Relationship Between Anemia and Subjective Symptoms in White-Collar Workers

**DOI:** 10.7759/cureus.83819

**Published:** 2025-05-09

**Authors:** Toshimitsu Iikuni, Kosuke Hotaka, Takashi Shimazaki, Takashi Yamauchi, Machi Suka

**Affiliations:** 1 Department of Public Health and Environmental Medicine, The Jikei University School of Medicine, Tokyo, JPN

**Keywords:** anemia, dizziness, dyspnea, edema, health checkup, subjective symptoms, workers

## Abstract

Introduction: Anemia has been shown to reduce workers’ performance and health. However, the relationship between anemia and subjective symptoms remains poorly understood. This study investigated the prevalence of anemia and subjective symptoms and the association between anemia and subjective symptoms among white-collar workers.

Methods: This study analyzed health examination data of Japanese white-collar workers (n= 103,530) from April 2023 to March 2024. The prevalence of three subjective symptoms, dizziness, palpitation/dyspnea, and edema, was compared among three categories without anemia (i.e., male hemoglobin value: 13 g/dL or female hemoglobin value: 12 g/dL), mild anemia (i.e., male hemoglobin value: 12-12.9 g/dL or female hemoglobin value: 11-11.9 g/dL), and moderate or severe anemia (i.e., male hemoglobin value: <12 g/dL or female hemoglobin value: <11 g/dL). To examine the association between anemia and three subjective symptoms, we performed multivariate logistic regression analysis with the presence or absence of subjective symptoms as the dependent variable. Additionally, a sex-based subgroup analysis was conducted.

Results: Among the participants, 5.1% had mild anemia, while 1.8% had moderate or severe anemia. Dizziness, palpitation/dyspnea, and edema were reported by 4.5%, 3.2%, and 7.4%, respectively. Anemia was significantly associated with all three subjective symptoms. Regarding dizziness, the multivariate odds ratio (OR) of mild anemia (OR: 1.28, 95%confidence interval, CI: 1.14-1.42) and moderate or severe anemia (OR: 1.56, 95% CI: 1.33-1.84) were >1. For palpitation and dyspnea, the multivariate OR of mild anemia (OR: 1.21, 95% CI: 1.05-1.39) and moderate or severe anemia (OR: 1.74, 95% CI: 1.44-2.11) were >1. Regarding edema, the multivariate OR of mild anemia (OR: 1.19, 95% CI: 1.09-1.30) and moderate or severe anemia (OR: 1.24, 95% CI: 1.09-1.43) were >1.

Conclusions:Anemia was associated with the three subjective symptoms even in mild conditions. These results may contribute to reduced work productivity, particularly through presenteeism and absenteeism. The study findings highlight the importance of early detection and treatment through health checkups, dietary interventions, and iron supplementation.

## Introduction

Anemia, a condition characterized by a reduced number of red blood cells or lower than normal hemoglobin concentration, impairs the blood’s ability to transport oxygen to the body’s tissues [1,2]. Anemia has been shown to reduce work productivity, leading to social and economic losses [1-3]. Additionally, research has linked anemia to health risks, including depression, heart failure, and dementia, highlighting the importance of early detection and effective treatment in occupational health settings [2,4-6].

Despite growing evidence of the adverse effects of anemia on workers' performance and health [7], the relationship between anemia and subjective symptoms remains poorly understood. Previous studies investigating this association reported inconclusive findings due to the limited quality of evidence [8,9]. One study was reported with a small sample size, highlighting a substantial limitation [8]. Another study revealed an association between subjective symptoms and anemia, but only in men [9], and its insufficient adjustment for confounding factors further weakened its validity [9]. Therefore, further research is needed to examine the association between anemia and subjective symptoms, particularly using large-scale data while accounting for potential confounding factors.

This study aimed to determine the prevalence of anemia and subjective symptoms, examine their association using data from a large cohort of white-collar workers in Tokyo, Japan, and highlight the importance of early detection and treatment of anemia.

## Materials and methods

Participants and study design

Electronic data on the health examinations conducted from April 2023 to March 2024 were collected from the Tokyo Health Service Association (Tokyo, Japan), where 130,000 employees across 1,400 companies undergo annual health examinations. The study protocol was approved by the ethics committee of the Tokyo Health Service Association (Tou-Yo-Rin No. 007, November 25, 2024) and followed the Ethical Guidelines for Medical and Biological Research Involving Human Subjects by the Japanese Government. This study adhered to the Strengthening the Reporting of Observational Studies in Epidemiology guidelines for cross-sectional studies (https://www.strobe-statement.org/index.php?id=strobe-home). The corresponding checklist is presented in Appendix 1.

A total of 123,024 patients with anemia underwent health examinations during the study period. To compare patients with anemia and those without anemia and a treatment intervention, we excluded 437 patients who had undergone anemia treatment within the past year. Additionally, 19,494 patients who did not respond to the five items required for the study, height, weight, blood pressure, hemoglobin level, and sleep quality, were excluded. Ultimately, 103,530 patients (59,864 men and 43,666 women) were included in the final analysis. The Tokyo Health Service Association obtained written informed consent from all health examinees, ensuring anonymity and confidentiality in data usage for research purposes.

Measure

Annual health examinations, including anthropometric measurements, laboratory tests, and self-administered questionnaires, were conducted following the Standard Health Examination Program by the Japanese Government [10]. Participants submitted the standard questionnaire at the reception desk on the day of the examination, where trained staff reviewed it for completion.

Weight (kg, measured to the nearest 0.1 kg) and height (cm, measured to the nearest 0.1 cm) were measured with the participants wearing light clothing and standing without shoes. Blood pressure was measured using an electronic sphygmomanometer, with the participants sitting on a chair after at least five minutes of rest. Blood samples were analyzed at a laboratory of the Tokyo Health Service Association, where both internal and external quality controls of the laboratory data are regularly in accordance with the guidelines of the expert committee for data standardization.

Anemia

Anemia was assessed using hemoglobin levels from health examination data. Hemoglobin cutoff between presence and absence of anemia was adopted, consistent with the World Health Organization’s cutoff for without anemia and mild anemia [11]. To comprehensively investigate the relation between anemia and subjective symptoms, we adopted the original reference hemoglobin cutoff of the Tokyo Health Service Association as a cutoff between mild anemia and moderate or severe anemia [12]. Participants were classified into three categories: without anemia (i.e., male hemoglobin value: 13 g/dL or female hemoglobin value: 12 g/dL) [11], mild anemia (i.e., male hemoglobin value: 12-12.9 g/dL or female hemoglobin value: 11-11.9 g/dL) [11,12], and moderate or severe anemia (i.e., male hemoglobin value: <12 g/dL, or female hemoglobin value: <11 g/dL) [11,12].

Subjective Symptoms

Participants were asked: “Do you currently experience any of the following subjective symptoms?” Those who reported experiencing at least one symptom were classified as having subjective symptoms. The assessed symptoms included fatigue, dizziness, headache, stiff shoulder, back pain, limb numbness, palpitation/dyspnea, irritability, and edema.

Attributes of Participants

To consider the participants’ attributes, we used sex, age, body mass index (BMI), blood pressure, and sleep quality from the health examination data.

Age group: The participants' age was categorized into the following five categories: ≤29 years, 30-39 years, 40-49 years, 50-59 years, and ≥60 years.

BMI: BMI was calculated based on height and weight and classified into the following three categories: <18.5, 18.5-25, and ≥25 kg/m^2^ [13].

Blood pressure: Blood pressure was classified into the following three groups: low blood pressure (i.e., systolic blood pressure: ≤100  mmHg or diastolic blood pressure: ≤60  mmHg), high blood pressure (i.e., systolic blood pressure: ≥140  mmHg or diastolic blood pressure: ≥90  mmHg), and normal blood pressure (i.e., not low blood pressure or high blood pressure) [10,14].

Sleep quality: Participants responded to the question: “Do you sleep well and get enough rest? (Yes/No).” Those who answered “No” were classified as experiencing inadequate sleep.

Medical History

Participants were asked: “Have you consulted a doctor or received medical treatment for the following diseases within the past year?” Those who answered “Yes” were classified as having a medical condition. We considered the following diseases: cardiovascular diseases, such as myocardial infarction, angina pectoris, arrhythmia, and valvular heart disease; kidney diseases, such as nephritis and renal failure; cerebrovascular diseases, such as cerebral infarction and cerebral hemorrhage; and uterine fibroids.

Statistical analysis

We conducted cross-tabulation and chi-square tests to examine the association between anemia and nine subjective symptoms (fatigue, dizziness, headache, stiff shoulder, back pain, limb numbness, palpitation/dyspnea, irritability, and edema), reporting the effect size and p values. Three subjective symptoms (dizziness, palpitation/dyspnea, and edema) were selected for further analysis owing to their high effect size and strong association with anemia. Appendix 2 presents the cross-tabulation and chi-square tests for the nine subjective symptoms.

To examine the association between anemia and the three representative subjective symptoms, we performed a multivariate logistic regression analysis with the presence or absence of subjective symptoms as the dependent variable. The demographic variables and other potential confounders (BMI, blood pressure, sleep quality, depressive symptoms, cardiovascular disease, kidney disease, cerebrovascular disease, and uterine fibroids) were adjusted.

As a subgroup analysis, sex-based multivariate logistic regression analysis was conducted to examine the association between anemia and subjective symptoms. A p value of <0.05 was considered statistically significant. All analyses were performed using Statistical Package for the Social Sciences version 29 software (IBM Corp., Chicago, IL).

## Results

Table 1 presents participants’ characteristics. Among all participants, 5.1% (n = 5,283) had mild anemia, with 77.7% (n = 4,105) being female participants. Additionally, 1.8% (n = 1,850) had moderate or severe anemia, with 84.4% (n = 1,561) being female participants. Those with dizziness were 4.5% (n = 4,691), and 68.7% (n = 3,222) were female participants. Palpitation/dyspnea was reported by 3.2% (n = 3,279), with 56.9% (n = 1,866) being female participants. Edema was observed in 7.4% (n = 7,683), with 79.9% (n = 6,137) of them being female participants.

**Table 1:** Characteristics of participants

Characteristics	Total (n = 103,530)	Male (n = 59,864), n (%)	Female (n = 43,666), n (%)
Anemia			
Without anemia	96,397	58,397 (97.5)	38,000 (87)
Mild anemia	5,283	1,178 (2)	4,105 (9.4)
Moderate or severe anemia	1,850	289 (0.5)	1,561 (3.6)
Subjective symptoms			
Dizziness	4,691	1,469 (2.5)	3,222 (7.4)
Palpitation/dyspnea	3,279	1,413 (2.4)	1,866 (4.3)
Edema	7,683	1,546 (2.6)	6,137 (14.1)
Age group			
≤29 years	17,752	8,089 (13.5)	9,663 (22.1)
30-39 years	23,069	13,105 (21.9)	9,964 (22.8)
40-49 years	23,152	13,720 (22.9)	9,432 (21.6)
50-59 years	24,902	15,863 (26.5)	9,039 (20.7)
≥60 years	14,655	9,087 (15.2)	5,568 (12.8)
Body mass index			
<18.5 kg/m^2^	10,149	2,525 (4.2)	7,624 (17.5)
18.5-25 kg/m^2^	68,175	38,825 (64.9)	29,350 (67.2)
≥25 kg/m^2^	25,206	18,514 (30.9)	6,692 (15.3)
Sleep quality			
Enough	67,289	40,157 (67.1)	27,132 (62.1)
Not enough	36,241	19,707 (32.9)	16,534 (37.9)
Blood pressure			
Normal blood pressure	65,772	40,389 (67.5)	25,383 (58.1)
Low blood pressure	18,953	5,390 (9)	13,563 (31.1)
High blood pressure	18,805	14,085 (23.5)	4,720 (10.8)
Depression			
Absence	102,282	59,110 (98.7)	43,172 (98.9)
Presence	1,248	754 (1.3)	494 (1.1)
Cardiovascular disease			
Absence	101,776	58,536 (97.8)	43,240 (99)
Presence	1,754	1,328 (2.2)	426 (1)
Kidney disease			
Absence	103,225	59,651 (99.6)	43,574 (99.8)
Presence	305	213 (0.4)	92 (0.2)
Cerebrovascular disease			
Absence	103,102	59,534 (99.4)	43,568 (99.8)
Presence	428	330 (0.6)	98 (0.2)
Uterine fibroids			
Absence	102,273	59,864 (100)	42,409 (97.1)
Presence	1,257	0 (0)	1,257 (2.9)

Tables 2-4 summarize the results of a multivariate logistic regression analysis of the association between anemia and subjective symptoms. Regarding dizziness, the multivariate odds ratio (OR) of mild anemia (OR: 1.28, 95% confidence interval, CI: 1.14-1.42) and moderate or severe anemia (OR: 1.56, 95% CI: 1.33-1.84) were >1. For palpitation and dyspnea, the multivariate OR of mild anemia (OR: 1.21, 95% CI: 1.05-1.39) and moderate or severe anemia (OR: 1.74, 95% CI: 1.44-2.11) were >1. Regarding edema, the multivariate OR of mild anemia (OR: 1.19, 95% CI: 1.09-1.30) and moderate or severe anemia (OR: 1.24, 95% CI: 1.09-1.43) were >1.

**Table 2:** Univariate and multivariate logistic regression analysis with the presence/absence of dizziness as the dependent variable

Dizziness	Total (n = 103,530), odds ratio (95% CI)		Subgroup analysis by sex, odds ratio (95% CI)		
			Male (n = 59,864), multivariate	Female (n = 43,666), multivariate	
	Univariate	Multivariate			
Anemia					
Without anemia	Reference	Reference	Reference	Reference	
Mild anemia	1.84 (1.66-2.05)	1.28 (1.14-1.42)	1.31 (0.95-1.81)	1.24 (1.11-1.40)	
Moderate or severe anemia	2.53 (2.17-2.95)	1.56 (1.33-1.84)	1.65 (0.97-2.83)	1.5 (1.27-1.78)	
Sex					
Male	Reference	Reference	-	-	
Female	3.17 (2.97-3.37)	2.94 (2.74-3.14)	-	-	
Age group					
≤29 years	Reference	Reference	Reference	Reference	
30-39 years	1.04 (0.94-1.14)	1.07 (0.97-1.18)	1.04 (0.85-1.29)	1.1 (0.98-1.23)	
40-49 years	1.21 (1.10-1.33)	1.2 (1.09-1.32)	1.29 (1.06-1.58)	1.19 (1.06-1.33)	
50-59 years	1.13 (1.03-1.24)	1.15 (1.04-1.27)	1.36 (1.12-1.66)	1.08 (0.96-1.22)	
≥60 years	0.92 (0.82-1.03)	0.997 (0.89-1.12)	1.37 (1.10-1.70)	0.86 (0.74-0.99)	
Body mass index					
<18.5 kg/m^2^	1.5 (1.37-1.63)	1.09 (0.997-1.16)	1.23 (0.95-1.58)	1.08 (0.98-1.19)	
18.5-25 kg/m^2^	Reference	Reference	Reference	Reference	
≥25 kg/m^2^	0.96 (0.90-1.03)	1.12 (1.03-1.21)	1.09 (0.97-1.22)	1.14 (1.02-1.26)	
Sleep quality					
Enough	Reference	Reference	Reference	Reference	
Not enough	3.31 (3.11-3.51)	3.06 (2.88-3.25)	3.95 (3.54-4.42)	2.72 (2.52-2.92)	
Blood pressure					
Normal blood pressure	Reference	Reference	Reference	Reference	
Low blood pressure	1.46 (1.36-1.57)	1.08 (0.997-1.16)	1.01 (0.83-1.21)	1.08 (0.99-1.17)	
High blood pressure	0.77 (0.71-0.85)	0.89 (0.81-0.97)	0.86 (0.76-0.98)	0.91 (0.80-1.03)	
Depression					
Absence	Reference	Reference	Reference	Reference	
Presence	3.65 (3.11-4.29)	3.17 (2.68-3.75)	3.42 (2.66-4.40)	2.95 (2.36-3.68)	
Cardiovascular disease					
Absence	Reference	Reference	Reference	Reference	
Presence	1.82 (1.53-2.18)	2.19 (1.82-2.63)	2.02 (1.57-2.60)	2.18 (1.65-2.89)	
Kidney disease					
Absence	Reference	Reference	Reference	Reference	
Presence	1.48 (0.94-2.33)	1.43 (0.89-2.28)	1.74 (0.94-3.20)	1.05 (0.50-2.21)	
Cerebrovascular disease					
Absence	Reference	Reference	Reference	Reference	
Presence	1.65 (1.14-2.38)	2.04 (1.39-2.99)	2.39 (1.51-3.78)	1.43 (0.73-2.80)	
Uterine fibroids					
Absence	Reference	Reference	-	Reference	
Presence	2.56 (2.13-3.07)	1.3 (1.08-1.57)	-	1.32 (1.09-1.59)	

**Table 3:** Univariate and multivariate logistic regression analysis with the presence/absence of palpitation/dyspnea as the dependent variable

Palpitation/dyspnea	Total (n = 103,530), odds ratio (95% CI)		Subgroup analysis by sex, odds ratio (95% CI)	
	Univariate	Multivariate	Male (n = 59,864), multivariate	Female (n = 43,666), multivariate
Anemia				
Without anemia	Reference	Reference	Reference	Reference
Mild anemia	1.46 (1.28-1.68)	1.21 (1.05-1.39)	1.44 (1.05-1.96)	1.15 (0.98-1.35)
Moderate or severe anemia	2.36 (1.96-2.84)	1.74 (1.44-2.11)	2.49 (1.58-3.93)	1.6 (1.30-1.98)
Sex				
Male	Reference	Reference	-	-
Female	1.85 (1.72-1.98)	1.86 (1.72-2.02)	-	-
Age group				
≤29 years	Reference	Reference	Reference	Reference
30-39 years	1.28 (1.13-1.46)	1.23 (1.08-1.40)	1.25 (0.98-1.58)	1.24 (1.06-1.46)
40-49 years	1.62 (1.43-1.84)	1.42 (1.25-1.61)	1.58 (1.26-1.98)	1.36 (1.16-1.59)
50-59 years	1.86 (1.64-2.10)	1.56 (1.37-1.77)	1.67 (1.33-2.08)	1.54 (1.31-1.80)
≥60 years	1.87 (1.63-2.13)	1.57 (1.36-1.81)	1.83 (1.44-2.32)	1.39 (1.16-1.67)
Body mass index				
<18.5 kg/m^2^	1.37 (1.23-1.53)	1.19 (1.06-1.33)	1.27 (0.97-1.66)	1.16 (1.02-1.32)
18.5-25 kg/m^2^	Reference	Reference	Reference	Reference
≥25 kg/m^2^	1.27 (1.17-1.37)	1.22 (1.12-1.33)	1.29 (1.15-1.44)	1.15 (1.01-1.31)
Sleep quality				
Enough	Reference	Reference	Reference	Reference
Not enough	3.59 (3.34-3.87)	3.3 (3.07-3.55)	3.51 (3.14-3.92)	3.15 (2.85-3.48)
Blood pressure				
Normal blood pressure	Reference	Reference	Reference	Reference
Low blood pressure	1.11 (1.02-1.22)	0.97 (0.88-1.07)	1.04 (0.85-1.27)	0.94 (0.84-1.05)
High blood pressure	1.07 (0.98-1.18)	1.06 (0.96-1.17)	1.08 (0.95-1.23)	1.03 (0.88-1.20)
Depression				
Absence	Reference	Reference	Reference	Reference
Presence	3.89 (3.25-4.66)	3.22 (2.67-3.88)	2.85 (2.16-3.76)	3.55 (2.75-4.59)
Cardiovascular disease				
Absence	Reference	Reference	Reference	Reference
Presence	5.45 (4.74-6.26)	5.35 (4.61-6.21)	4.66 (3.84-5.64)	6.42 (5.04-8.18)
Kidney disease				
Absence	Reference	Reference	Reference	Reference
Presence	1.92 (1.19-3.10)	1.46 (0.89-2.40)	1.45 (0.78-2.69)	1.18 (0.50-2.78)
Cerebrovascular disease				
Absence	Reference	Reference	Reference	Reference
Presence	1.58 (1.02-2.46)	1.28 (0.81-2.03)	1.17 (0.66-2.07)	1.46 (0.68-3.15)
Uterine fibroids				
Absence	Reference	Reference	-	Reference
Presence	2.72 (2.22-3.35)	1.63 (1.31-2.02)	-	1.64 (1.32-2.03)

**Table 4:** Univariate and multivariate logistic regression analysis with the presence/absence of edema as the dependent variable

Edema	Total (n = 103,530), odds ratio (95% CI)		Subgroup analysis by sex, odds ratio (95% CI)		
			Male (n = 59,864), multivariate	Female (n = 43,666), multivariate	
	Univariate	Multivariate			
Anemia					
Without anemia	Reference	Reference	Reference	Reference	
Mild anemia	1.95 (1.79-2.12)	1.19 (1.09-1.30)	2.52 (1.97-3.22)	1.05 (0.96-1.16)	
Moderate or severe anemia	2.38 (2.09-2.71)	1.24 (1.09-1.43)	3.9 (2.65-5.73)	1.04 (0.90-1.21)	
Sex					
Male	Reference	Reference	-	-	
Female	6.17 (5.83-6.53)	6.49 (6.10-6.90)	-	-	
Age group					
≤29 years	Reference	Reference	Reference	Reference	
30-39 years	0.96 (0.89-1.03)	1.01 (0.93-1.09)	1.54 (1.19-1.99)	1.02 (0.94-1.10)	
40-49 years	0.95 (0.88-1.02)	0.95 (0.88-1.03)	1.96 (1.52-2.51)	0.91 (0.84-0.99)	
50-59 years	0.9 (0.84-0.97)	0.94 (0.87-1.01)	2.57 (2.02-3.27)	0.79 (0.72-0.86)	
≥60 years	0.71 (0.65-0.78)	0.77 (0.70-0.85)	2.9 (2.25-3.75)	0.54 (0.48-0.60)	
Body mass index					
<18.5 kg/m^2^	1.15 (1.07-1.24)	0.7 (0.65-0.76)	0.72 (0.51-1.03)	0.69 (0.63-0.75)	
18.5-25 kg/m^2^	Reference	Reference	Reference	Reference	
≥25 kg/m^2^	1.09 (1.03-1.15)	1.53 (1.44-1.63)	1.92 (1.73-2.14)	1.37 (1.27-1.48)	
Sleep quality					
Enough	Reference	Reference	Reference	Reference	
Not enough	2.91 (2.78-3.05)	2.76 (2.62-2.90)	3.48 (3.13-3.87)	2.61 (2.47-2.76)	
Blood pressure					
Normal blood pressure	Reference	Reference	Reference	Reference	
Low blood pressure	1.61 (1.53-1.70)	1.1 (1.04-1.17)	0.99 (0.80-1.21)	1.06 (0.995-1.13)	
High blood pressure	0.79 (0.74-0.85)	0.95 (0.88-1.02)	0.99 (0.88-1.12)	0.9 (0.82-0.997)	
Depression					
Absence	Reference	Reference	Reference	Reference	
Presence	1.77 (1.49-2.10)	1.51 (1.26-1.81)	1.1 (0.75-1.60)	1.66 (1.34-2.05)	
Cardiovascular disease					
Absence	Reference	Reference	Reference	Reference	
Presence	1.28 (1.08-1.50)	1.82 (1.53-2.17)	1.68 (1.32-2.13)	1.48 (1.14-1.91)	
Kidney disease					
Absence	Reference	Reference	Reference	Reference	
Presence	2.7 (2.01-3.62)	3.54 (2.57-4.89)	2.87 (1.85-4.46)	2.54 (1.59-4.06)	
Cerebrovascular disease					
Absence	Reference	Reference	Reference	Reference	
Presence	1.18 (0.84-1.66)	1.74 (1.22-2.50)	1.82 (1.16-2.86)	1.24 (0.70-2.18)	
Uterine fibroids					
Absence	Reference	Reference	-	Reference	
Presence	3.14 (2.72-3.61)	1.34 (1.15-1.55)	-	1.38 (1.19-1.59)	

The sex-based subgroup analysis revealed a differential association between anemia and subjective symptoms. The prevalence of dizziness was higher in women, whereas palpitation, dyspnea, and edema were frequently observed in men.

## Discussion

Summary of the results

This study examined the prevalence of anemia and its subjective symptoms among white-collar workers in Tokyo, Japan. The other national data of the health examination for workers in Japan conducted from April 2023 to June 2023 reported that the prevalence of anemia was 8.6%, which was approximately consistent with the present study finding [15]. The multivariate logistic regression analysis for considering the relationship between anemia and subjective symptoms showed that anemia was significantly associated with dizziness, palpitation/dyspnea, and edema, even with mild anemia. In the sex-based subgroup analysis, anemia was strongly associated with dizziness in female participants compared with male participants, although the difference was marginal. Conversely, male participants with anemia exhibited a stronger association with palpitation, dyspnea, and edema than female participants.

Possible explanation of the relationship between anemia and subjective symptoms

Among the white-collar workers in Tokyo, anemia was strongly correlated with dizziness, palpitation/dyspnea, and edema. Previous studies have demonstrated associations between anemia and dizziness, palpitation/dyspnea, and edema, supporting the validity of our results [16]. In previous studies, relatively little was known about the subjective symptoms of anemia. This study was the first to examine the association between anemia and subjective symptoms using a large sample of white-collar workers with sophisticated multivariate analysis.

Additionally, a key finding from our study is that subjective symptoms occur even in mild anemia, which is typically not considered necessary to treat. According to previous studies, the subjective symptoms, including dizziness and palpitation/dyspnea, reduce productivity [17,18]. Additionally, these subjective symptoms may contribute to presenteeism (i.e., working less effectively) and absenteeism (i.e., being out of work), both of which have broader implications for employee health worldwide [18-20]. Therefore, the early detection and treatment of anemia may contribute to improving subjective symptoms and occupational health in white-collar workers. Promoting health check-ups is a serious issue of health policy worldwide. According to the Organization for Economic Co-operation and Development (OECD) report, only three countries (France, Japan, and Korea) regularly require the provision of health check-ups to employees across sectors among OECD member countries [21]. Similarly, encouraging early intervention for anemia may help improve workers’ health conditions. Dietary interventions have been shown to improve blood conditions [22]. Dietary therapy, which includes increasing the intake of iron and vitamin C, contributes to improving anemia [23]. Vitamin C is an essential enhancer of iron absorption; therefore, workers who have anemia should increase their intake of iron and vitamin C concurrently [24,25]. Furthermore, iron supplementation is recommended if anemia progresses [26].

Sex differences in the prevalence of subjective symptoms of anemia

The analysis of sex differences in subjective symptoms of anemia revealed that dizziness was frequent among female participants compared with male participants. A previous study consistently reported a higher prevalence of moderate or severe dizziness or vertigo in women than in men [15,27,28]. This difference may be attributed to various factors, including the influence of psychological function associated with reproductive role, a higher tendency to selectively attend to body cues, and a greater willingness to report the perceived symptoms [29].

Conversely, palpitation, dyspnea, and edema were more frequent among male participants. This discrepancy may stem from the higher prevalence of cardiovascular diseases or kidney diseases in male participants, both of which cause these symptoms. However, this study did not adequately adjust for cardiovascular diseases or kidney diseases; therefore, male participants might have more frequently complained of palpitations, dyspnea, and edema due to their past medical history. To investigate why male participants with anemia were more strongly associated with edema, we reanalyzed the data by comparing the data between male and female participants; male edema was associated with a history of diabetes mellitus (data not shown). Edema is caused by diabetic nephropathy, which is caused by progressive diabetes mellitus [30]. Additionally, it is possible that the eating and exercise habits of male participants with diabetes mellitus affect edema. Thus, the association between male anemia and edema may have been confounded by a history of diabetes mellitus. Therefore, this study’s subgroup analysis could not conclusively establish a sex difference in the association between anemia and subjective symptoms.

Strengths and limitations

This study had three strengths. First, this study was very large, with 103,530 participants. Because of the large number of participants, the estimated accuracy improved, and highly reliable results were obtained. Second, we considered more confounding factors that were highly associated with subjective symptoms [8,9]. We analyzed potential confounding factors, such as BMI, sleep quality, and major diseases, that were likely to be associated with subjective symptoms, and we obtained highly valid results. Third, anemia was stratified into mild and moderate or severe anemia, and results showed that even mild anemia was significantly associated with subjective symptoms, which are dizziness, palpitation/dyspnea, and edema, despite considering confounding factors that were highly associated with subjective symptoms.

However, this study had some limitations. First, the participants were limited to white-collar workers in Tokyo. Therefore, the applicability of our findings to the blue-collar worker was unknown. Second, we could not completely adjust for confounding factors such as stress levels, diet, chronic diseases, or medication use because our data were limited to electronic records of health examinations collected from the Tokyo Health Service Association. Additionally, undiagnosed conditions could not be classified as the presence of disease. Third, regarding the sex-based subgroup analysis, we could not sufficiently conclude a sex difference in the association between anemia and subjective symptoms. Fourth, the cross-sectional nature of this study made it difficult to determine causality between anemia and subjective symptoms. Further studies need to be conducted on longitudinal or intervention studies.

## Conclusions

To the best of our knowledge, this is the first to examine the association between anemia and subjective symptoms, particularly using large-scale data while accounting for potential confounding factors. Among the white-collar workers in Tokyo, anemia was associated with subjective symptoms (i.e., dizziness, palpitation/dyspnea, and edema) even in the mild anemia that does not require treatment. Addressing anemia may also help prevent presenteeism and absenteeism. Therefore, early detection and dietary interventions are important for workers' health and productivity. Further research is required to consider the sex difference in the association between anemia and subjective symptoms.

## Appendices

Appendix 1

**Table 5:** Checklist of items that should be included in reports of cross-sectional studies ^*^Give information separately for exposed and unexposed groups

Items	Item no.	Recommendation	Page no.
Title and abstract	1	(a) Indicate the study’s design with a commonly used term in the title or the abstract	1, 2
		(b) Provide in the abstract an informative and balanced summary of what was done and what was found	2
Introduction			
Background/rationale	2	Explain the scientific background and rationale for the investigation being reported	4
Objectives	3	State-specific objectives, including any prespecified hypotheses	4
Methods			
Study design	4	Present key elements of the study design early in the paper	4, 5
Setting	5	Describe the setting, locations, and relevant dates, including periods of recruitment, exposure, follow-up, and data collection	4, 5
Participants	6	Give the eligibility criteria, and the sources and methods of selection of participants	4, 5
Variables	7	Clearly define all outcomes, exposures, predictors, potential confounders, and effect modifiers. Give diagnostic criteria, if applicable	5-7
Data sources/measurement	8^*^	For each variable of interest, give sources of data and details of methods of assessment (measurement). Describe the comparability of assessment methods if there is more than one group	5-7
Bias	9	Describe any efforts to address potential sources of bias	5
Study size	10	Explain how the study size was arrived at	4, 5
Quantitative variables	11	Explain how quantitative variables were handled in the analyses. If applicable, describe which groupings were chosen and why	7, 8
Statistical methods	12	(a) Describe all statistical methods, including those used to control for confounding	7, 8
		(b) Describe any methods used to examine subgroups and interactions	7, 8
		(c) Explain how missing data were addressed	7, 8
		(d) If applicable, describe analytical methods taking account of sampling strategy	7, 8
		(e) Describe any sensitivity analyses	7, 8
Results			
Participants	13^*^	(a) Report numbers of individuals at each stage of study, e.g., numbers potentially eligible, examined for eligibility, confirmed eligible, included in the study, completing follow-up, and analyzed	8
		(b) Give reasons for nonparticipation at each stage	8
		(c) Consider the use of a flow diagram	8
Descriptive data	14^*^	(a) Give characteristics of study participants (e.g., demographic, clinical, and social) and information on exposures and potential confounders	8
		(b) Indicate the number of participants with missing data for each variable of interest	8
Outcome data	15^*^	Report numbers of outcome events or summary measures	8, 9
Main results	16	(a) Give unadjusted estimates and, if applicable, confounder-adjusted estimates and their precision (e.g., 95% confidence interval). Make clear which confounders were adjusted for and why they were included	8, 9
		(b) Report category boundaries when continuous variables were categorized	8, 9
		(c) If relevant, consider translating estimates of relative risk into absolute risk for a meaningful time period	8, 9
Other analyses	17	Report other analyses done, e.g., analyses of subgroups and interactions, and sensitivity analyses	9
Discussion			
Key results	18	Summarize key results with reference to study objectives	9
Limitations	19	Discuss limitations of the study, taking into account sources of potential bias or imprecision. Discuss both the direction and magnitude of any potential bias	12
Interpretation	20	Give a cautious overall interpretation of results, considering objectives, limitations, multiplicity of analyses, results from similar studies, and other relevant evidence	9-11
Generalizability	21	Discuss the generalizability (external validity) of the study results	12
Other information			
Funding	22	Give the source of funding and the role of the funders for the present study and, if applicable, for the original study on which the present article is based	12

Appendix 2

**Table 6:** Characteristics of participants by subjective symptoms

Subjective symptoms	Total (n = 103,530)					Subgroup analysis by sex									
						Male (n = 59,864)					Female (n = 43,666)				
	Without anemia (n = 96,397), n (%)	Mild anemia (n = 5,283), n (%)	Moderate or severe anemia (n = 1,850), n (%)	Chi-square test	Effect size	Without anemia (n = 58,397), n (%)	Mild anemia (n = 1,178), n (%)	Moderate or severe anemia (n = 289), n (%)	Chi-square test	Effect size	Without anemia (n = 38,000), n (%)	Mild anemia (n = 4,105), n (%)	Moderate or severe anemia (n = 1,561), n (%)	Chi-square test	Effect size
Tired easily	11,782 (12.2)	762 (14.4)	337 (18.2)	p < 0.001	0.028	5,913 (10.1)	142 (12.1)	38 (13.1)	p = 0.023	0.011	5,869 (15.4)	620 (15.1)	299 (19.2)	p < 0.001	0.019
Dizziness	4,104 (4.3)	400 (7.6)	187 (10.1)	p < 0.001	0.05	1,411 (2.4)	42 (3.6)	16 (5.5)	p < 0.001	0.017	2,693 (7.1)	358 (8.7)	171 (11.0)	p < 0.001	0.032
Headache	7,286 (7.6)	572 (10.8)	225 (12.2)	p < 0.001	0.035	2,825 (4.8)	61 (5.2)	6 (2.1)	p = 0.079	0.009	4,461 (11.7)	511 (12.4)	219 (14.0)	p = 0.012	0.014
Stiff shoulder	26,606 (27.6)	1,779 (33.7)	633 (34.2)	p < 0.001	0.035	12,118 (20.8)	261 (22.2)	54 (18.7)	p = 0.341	0.006	14,488 (38.1)	1,518 (37.0)	579 (37.1)	p = 0.271	0.008
Back pain	13,231 (13.7)	808 (15.3)	285 (15.4)	p < 0.001	0.012	6,935 (11.9)	160 (13.6)	40 (13.8)	p = 0.121	0.008	6,296 (16.6)	648 (15.8)	245 (15.7)	p = 0.309	0.007
Numbness in limbs	3,819 (4)	248 (4.7)	96 (5.2)	p < 0.001	0.011	2,161 (3.7)	78 (6.6)	25 (8.7)	p < 0.001	0.028	1,658 (4.4)	170 (4.1)	71 (4.5)	p = 0.743	0.004
Palpitation/dyspnea	2,921 (3)	231 (4.4)	127 (6.9)	p < 0.001	0.033	1,343 (2.3)	46 (3.9)	24 (8.3)	p < 0.001	0.031	1,578 (4.2)	185 (4.5)	103 (6.6)	p < 0.001	0.023
Irritated easily	10,681 (11.1)	736 (13.9)	300 (16.2)	p < 0.001	0.029	4,977 (8.5)	111 (9.4)	23 (8.0)	p = 0.516	0.005	5,704 (15.0)	625 (15.2)	277 (17.7)	p = 0.012	0.014
Edema	6,730 (7)	673 (12.7)	280 (15.1)	p < 0.001	0.063	1,432 (2.5)	77 (6.5)	37 (12.8)	p < 0.001	0.057	5,298 (13.9)	596 (14.5)	243 (15.6)	p = 0.130	0.01
